# Metabolic Engineering of *Klebsiella pneumoniae* for the Production of 2-Butanone from Glucose

**DOI:** 10.1371/journal.pone.0140508

**Published:** 2015-10-14

**Authors:** Zhen Chen, He Sun, Jinhai Huang, Yao Wu, Dehua Liu

**Affiliations:** 1 Institute of Applied Chemistry, Department of Chemical Engineering, Tsinghua University, Beijing 100084, China; 2 Tsinghua Innovation Center in Dongguan, Dongguan 523808, China; Tsinghua University, CHINA

## Abstract

2-Butanone is an important commodity chemical of wide application in different areas. In this study, *Klebsiella pneumoniae* was engineered to directly produce 2-butanone from glucose by extending its native 2, 3-butanediol synthesis pathway. To identify the potential enzyme for the efficient conversion of 2, 3-butanediol to 2-butanone, we screened different glycerol dehydratases and diol dehydratases. By introducing the diol dehydratase from *Lactobacillus brevis* and deleting the *ldhA* gene encoding lactate dehydrogenase, the engineered *K*. *pneumoniae* was able to accumulate 246 mg/L of 2-butanone in shake flask. With further optimization of culture condition, the titer of 2-butanone was increased to 450 mg/L. This study lays the basis for developing an efficient biological process for 2-butanone production.

## Introduction

Metabolic engineering of industrial microorganisms for the production of valuable commodity chemicals using renewable biofeedstocks instead of non-renewable fossil resource is an important route towards sustainable bioeconomy. Recent development of metabolic engineering and related disciplines such as systems biology and synthetic biology has significantly accelerated the process of strain development for the production of both natural and non-natural products, e.g. 1, 3-propanediol [1] and 1, 4-butanediol [2]. Although significant advancement has been achieved, production of non-natural metabolites is still highly challenging mostly due to a lack of natural pathway and corresponding natural enzymes [3–5]. In this study, we reported the construction of a recombinant *Klebsiella pneumoniae* for the de novo production of 2-butanone from glucose by a non-natural pathway.

2-Butanone, also known as methyl ethyl ketone (MEK), is an important chemical which has been widely used in industry as solvent and plastic welding agent. 2-Butanone can also be used as a potential biofuel because it has high energy density (31.5 MJ/kg) and low latent heat of vaporization (0.44 MJ/Kg)[6]. There is no natural metabolic pathway for the production of 2-butanone. It was proposed that 2-butanone can be obtained by dehydration of bio-based 2, 3-butanediol [7]. 2, 3-Butanediol is a natural metabolite that can be produced from glucose by several microorganisms such as *Klebsiella pneumoniae* [8–10]. Dehydration of 2, 3-butanediol to 2-butanone can be achieved by chemical catalysis [7]. However, there is no natural enzyme which can specifically catalyze the dehydration of 2, 3-butanediol. It has been reported that meso-2, 3-butanediol can be used as a poor substrate of some diol dehydratases [11]. Thus, it is possible to directly produce 2-butanone from glucose by extending the metabolic synthetic pathway of 2, 3-butanediol. Recently, this proposed 2-butanone biosynthetic pathway was established in *Escherichia coli* [12] and *Saccharomyces cerevisiae* [13]. However, the production of 2-butanone was extremely low. Both *E*. *coli* and *S*. *cerevisiae* cannot naturally synthesize 2, 3-butanediol. Thus, a heterologous 2, 3-butanediol pathway has to be introduced and systematically optimized. Moreover, both *E*. *coli* and *S*. *cerevisiae* cannot produce coenzyme B12, an essential cofactor of diol dehydratase and glycerol dehydratase. Thus, this expensive coenzyme should be additionally added during the cultivation which would increase the cost of whole process.

**Fig 1:**
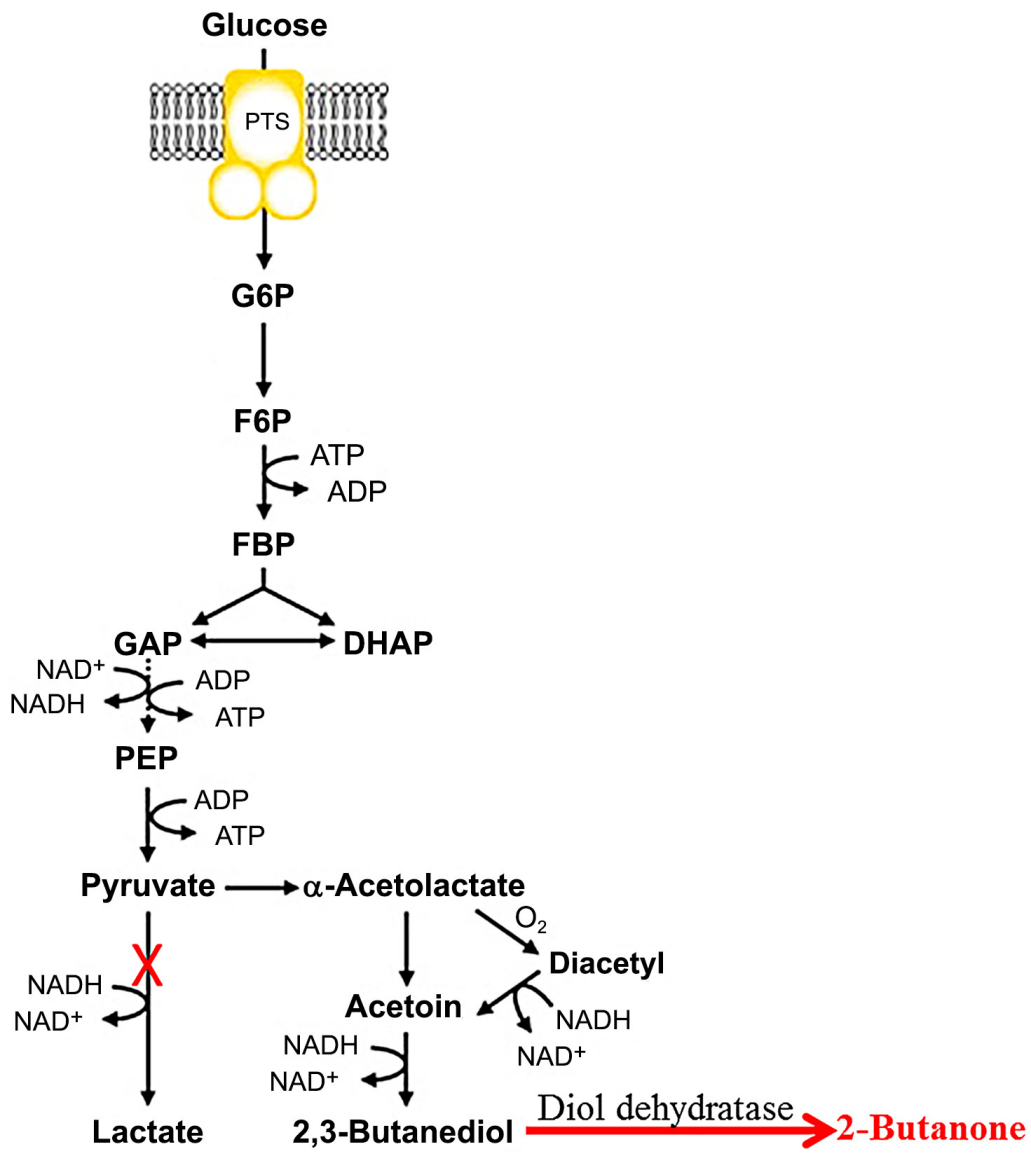
Metabolic pathway for 2-butanone production in *Klebsiella pneumoniae*.

In this study, we constructed and optimized the artificial 2-butanone synthetic pathway in *K*. *pneumoniae* (Fig 1). *K*. *pneumoniae* is a natural hyperproducer of 2, 3-butanediol which can accumulate meso-2, 3-butanediol up to 100g/L [8,14]. *K*. *pneumoniae* can also produce coenzyme B12, which make it an ideal chassis for 2-butanone production. In this study, we firstly screened different glycerol/diol dehydratases in order to identify the potential enzymes for the dehydration of 2,3-butanediol. The flux to 2-butanone synthesis was further enhanced by knocking out the *ldhA* gene encoded lactate dehydrogenase. By combination of process optimization, we were able to produce 450 mg/L 2-butanone from glucose. To the best of our knowledge, this is the highest titer that so far has been reported.

A diol dehydratase was introduced to convert the meso-2, 3-butanediol to 2-butanone. LdhA gene encoding lactate dehydrogenase was knocked out in order to enhance the flux from pyruvate to 2-butanone.

## Materials and Methods

### Bacterial strains and plasmids

Strains used in this study are listed in Table 1. *E*. *coli* DH5α was routinely used for the construction of plasmids and *E*. *coli* BL21 (DE3) was used for enzyme overexpression. *K*. *pneumoniae* HR526 (CGMCC 1.9131) was a high 1, 3-propanediol and 2, 3-butanediol producing strain. *K*. *pneumoniae* LDH526 was a mutant strain of *K*. *pneumoniae* HR526 with a deletion of *ldhA* gene [15].

**Table 1:** Strains used in this study.

Strains	Description	Source or reference
*E*. *coli* Strains		
DH5α	Host for plasmid construction	TIANGEN BIOTECH
BL21 (DE3)	Host for enzyme expression	TIANGEN BIOTECH
*K*. *pneumoniae* Strains		
HR526	Wildtype strain, CGMCC 1.9131	Xu et al., 2009
LDH526	HR526 with the deletion of *ldhA* gene	Xu et al., 2009
HR526/pHSG-kopdu	HR526 with the overexpression of diol dehydratase and its activator (*pduCDEGH*) from *K*. *oxytoca*	This study
HR526/pHSG-lbpdu	HR526 with the overexpression of diol dehydratase and its activator (*pduCDEGH*) from *L*. *brevis*	This study
HR526/pHSG-sepdu	HR526 with the overexpression of diol dehydratase and its activator (*pduCDEGH*) from *S*. *enterica*	This study
LDH526/pHSG-lbpdu	LDH526 with the overexpression of diol dehydratase and its activator (*pduCDEGH*) from *L*. *brevis*	This study

To construct plasmids for enzyme overexpression in *E*. *coli* BL21 (DE3), the genes encoding glycerol dehydratases (*gldABC*) or diol dehydratases (*pduCDE*) were amplified by PCR from genomic DNA. All of the forward primers contained an overlapped 25-nt sequence with pET28a at 5' ends (5'-GCAAATGGGTCGCGGATCCGAATTC-3'). Similarly, all of the reverse primers also contained an overlapped 25-nt sequences with pET28a at 5' ends (5'- GGTGCTCGAGTGCGGCCGCAAGCTT-3'). The amplified PCR fragments were inserted into plasmid pET28a (Novagen) digested with *Eco*RI and *Hin*dIII by Gibson assembly. The primers used herein are listed in S1 Table.

To construct plasmids for enzyme overexpression in *K*. *pneumoniae*, the genes encoding diol dehydratases and their activators (*pduCDEGH*) were amplified by PCR from genomic DNA. The forward primers contained an overlapped 20-nt sequence with pHSG298 at 5' ends (5'-CTATGACATGATTACGAATT-3'). The reverse primers also contained an overlapped 20-nt sequences with pHSG298 at 5' ends (5'- TGCCTGCAGGTCGACTCTAG-3'). The amplified PCR fragments were inserted into plasmid pHSG298 (TAKARA) digested with *Eco*RI and *Xba*I by Gibson assembly. The primers used herein are listed in S2 Table. The sequence accession numbers of glycerol dehydratases and diol dehydratases used in this study are listed in S3 Table.

### Protein expression and purification

Glycerol dehydratases and diol dehydratases were expressed in *E*. *coli* BL21 (DE3). The recombinant cells were first grown in LB media supplemented with 50 μg/ml kanamycin at 37°C until the OD_600_ reached 0.6. Isopropyl β-D-thiogalactopyranoside (IPTG) was then added to the final concentration of 0.1mM to induce the enzyme overexpression and the cultivation was continued for an additional 12-14h at 20°C. The cells were harvested, washed and suspended in the buffer consisting of 50 mM Na_2_HPO_4_ (pH 7.5), 0.5 M KCl, and 0.1 mM dithiothreitol. Suspended cells were disrupted by sonication and centrifuged at 10,000×g for 1h. The supernatant was purified by Ni^2+^-NTA column (GE Healthcare Bio-Sciences, Piscataway, NJ) to obtain the samples for activity assay. The purity of the enzymes was checked by SDS-PAGE and the protein concentrations were quantified using a Bio-Rad protein assay kit (Bio-Rad Laboratories).

### Enzyme assay of glycerol dehydratase and diol dehydratase

The determination of glycerol or diol dehydratase activity was based on the derived reaction of aldehydes/ketone with 3-methyl-2-benzothiazolinone hydrazone (MBTH) [16]. The assay mixture contained 200 μl of 40 mM HEPES (pH 8.0), 0.012 mM coenzyme B12, 50 mM KCl, 100 mM substrate (1, 2-propanediol or 2, 3-butanediol), and appropriate amount of enzyme. After incubation at 37°C for 1 min, the enzyme reaction was terminated by addition of 100 μl of MBTH solution (28 mM MBTH in 375 mM glycine buffer (pH 2.7)). After incubation at 100°C for 3 min, the samples were cooled on ice and 1 ml of FeCl_3_ solution (12.2 mM) was added. The mixtures were incubated at room temperature for 15 min and the amount of aldehyde formed was determined at 670 nm. Kinetic constants were obtained from non-linear regression data analysis and expressed as the mean ± S.D. All of the measures were repeated in triplicates.

### Cultivation and metabolite analysis

For the characterization of 2-butanone production by *K*. *pneumonia* mutants, cell cultivations were carried out in shake flasks at 37°C, 250 rpm for 36 h. The medium was composed of 60 g L^-1^ glucose, 7 g L^-1^ (NH_4_)_2_SO_4_, 6 g L^-1^ yeast extract, 0.2 g L^-1^ MgSO_4_.7H_2_0, 0.4 g L^-1^ KCl, 5 g L^-1^ KH_2_PO_4_, 0.02 g L^-1^ FeSO_4_.7H_2_0, 0.02 g L^-1^ MnSO_4_.7H_2_0, 0.02 g L^-1^ ZnSO_4_.7H_2_0, 50 mg L^-1^ kanamycin, 30 g L^-1^ CaCO_3_. When necessary, 1 μM coenzyme B12 was added. The fermentations were repeated in triplicates.

Quantification of 2-butanone, glucose and other organic acids were carried out by using High performance liquid chromatography (HPLC) as described before [15,17]. Cell concentration was determined at an optical density of 600nm and by dry biomass measurements.

## Results

### Characterization of glycerol dehydratases and diol dehydratases for the dehydratation of 2, 3-butanediol

So far, there is no known natural enzyme which can specifically catalyze the dehydration of 2, 3-butanediol. Thus, we tried to explore the promiscuous activity of natural dehydratases. Glycerol dehydratase and diol dehydratase can efficiently utilize both glycerol and 1, 2-propanediol as substrates. Concerning that 2, 3-butanediol is a structural analogue of glycerol and 1, 2-propanediol, we screened different glycerol dehydratases and diol dehydratases to evaluate their potential for 2, 3-butanediol dehydration. Three glycerol dehydratases and three diol dehydratase were evaluated, including glycerol dehydratase from *K*. *pneumoniae* MGH78578 (KPGDH), glycerol dehydratase from *Citrobacter freundii* ATCC 29229 (CFGDH), glycerol dehydratase from *Lactobacillus reuteri* DSM20016 (LRGDH), diol dehydratase from *Klebsiella oxytoca* M5al (KODDH), diol dehydratase from *Lactobacillus brevis* ATCC 367 (LBDDH), and diol dehydratase from *Salmonella enterica* ATCC 700720 (SEDDH). These enzymes have been previously shown to be efficiently catalyze the dehydration of glycerol or 1, 2-propanediol. However, their capabilities for the catalysis of 2,3-butanediol dehydration have not been explored. The corresponding genes were cloned into pET-28a and overexpressed in *E*. *coli*. The enzymes were purified and characterized. All of glycerol dehydratases and diol dehydratases can efficiently utilize 1, 2-propanediol as substrates (Table 2). The enzymes can also utilize meso-2, 3-butanediol as a week substrate. Generally, the *K*_*m*_ value is about 15–20 times higher than that of 1, 2-propanediol while the *K*_*cat*_ is about 10-fold lower than that of 1, 2-propanediol. The catalytic efficiency of meso-2, 3-butanediol is about 2-orders lower than of 1, 2-propanediol. When (2S, 3S)-2, 3-butanediol or (2R, 3R)-2, 3-butanediol was used as substrate, no enzyme activity was detected. This was consistent with the previous report that only meso-2, 3-butanediol can be dehydrated by glycerol dehydratases and diol dehydratases [11]. As illustrated in Table 2, the diol dehydratases showed better catalytic efficiency of meso-2, 3-butanediol than glycerol dehydratases. Thus, the three diol dehydratases were further introduced into *K*. *pneumoniae* to test their effect for 2-butanone production.

**Table 2:** Enzyme kinetics of glycerol dehydratases and diol dehydratases.

Enzyme	Substrate	*K*_*m*_	*K*_*cat*_	*K*_*cat*_ */K*_*m*_
		*mM*	*s* ^*-1*^	*mM* ^*-1*^ *s* ^*-1*^
KODDH	1,2-PDO	0.4± 0.1	313 ± 12	823± 24
	2,3-BDO	12.4± 2.2	28 ± 16	2.3 ± 0.2
LBDDH	1,2-PDO	0.4 ± 0.1	378 ± 11	879 ± 22
	2,3-BDO	10.4 ± 1.2	35 ± 11	3.4 ± 0.2
SEDDH	1,2-PDO	0.5± 0.1	294 ± 9	544 ± 18
	2,3-BDO	13.2± 2.8	22 ± 4	1.7 ± 0.2
KPGDH	1,2-PDO	1.1± 0.1	280 ±36	260 ±32
	2,3-BDO	22.2 ± 2.4	16 ± 5	0.7 ± 0.2
CFGDH	1,2-PDO	1.4 ± 0.1	289 ± 37	202 ±37
	2,3-BDO	16.2 ± 1.2	17 ± 11	1.1 ± 0.3
LRGDH	1,2-PDO	0.8 ± 0.1	228 ± 45	272 ± 352
	2,3-BDO	18.4 ± 1.4	20 ± 8	1.1 ± 0.4

All of 2, 3-butanediol used in this table is meso-2, 3-butanediol. KODDH: Diol dehydratase from *K*. *oxytoca*; LBDDH: Diol dehydratase from *L*. *brevis*; SEDDH: Diol dehydratase from *S*. *enterica*; KPGDH: Glycerol dehydratase from *K*. *pneumoniae*; CFGDH: Glycerol dehydratase from *C*. *freundii*; LRGDH: Glycerol dehydratase from *L*. *reuteri*.

### Overexperssion of diol dehydratases for 2-butanone production in *Klebsiellia pneumoniae*

The three diol dehydratasses as well as their activators were cloned into pHSG298 vector (TAKARA) and the corresponding plasmids were introduced into *K*. *pneumoniae* HR526 (CGMCC 1.9131) by electroporation. *K*. *pneumoniae* HR526 is a high 1, 3-propanediol producer when glycerol was used as substrate [15]. When glucose is used as substrate, meso-2, 3-butanediol become the major product. Although *K*. *pneumonia* HR526 possesses inherent glycerol dehydratase and diol dehydratase, no activity towards 2, 3-butanediol dehydration was detected. The strain also did not produce 2-butanone when glucose was used as substrate (Table 3). The structural genes of glycerol dehydratase and diol dehydratase of *K*. *pneumonia* are located within *dha* operon and *pdu* operon, respectively. The transcription of inherent *dha* operon and *pdu* operon is only induced by dihydroxyacetone (DHA) and 1, 2-propaediol, respectively [18]. When glucose was used as substrate, the *dha* and *pdu* operon cannot be transcribed. Interestingly, even with glycerol as substrate, there is no detectable accumulation of 2-butanone (data not shown). This is propably due to that glycerol is a much preferrable substrate of glycerol dehydratase and the dehydration of 2, 3-butaneediol is competitively inhibited when glycerol exists. All of the heterologous diol dehydratses and their activators can be well overexpressed in *K*. *pneumonia* HR526. The enzyme activities (towards 2, 3-butanediol dehydration) of *K*. *pneumonia* HR526 harboring KODDH, LBDDH and SEDDH were 0.35 U/mg, 0.28 U/mg and 0.21 U/mg, respectively. All of the mutants can produce 2-butanone (Table 3). Specifically, the highest 2-butanone accumulation (172 ± 23 mg/L) was obtained when *L*. *brevis* (LBDDH) was overexpressed. The residual 2, 3-butanediol is higher than 17 g/L, indicating that the 2, 3-butanediol is not the limiting factor for the low conversion yield. Besides 2, 3-butanediol, the main byproducts were lactate and acetoin (Table 3).

**Table 3:** 2-Butanone production by introducing different diol dehydratases into *K*. *peneumoniae* HR526.

*K*. *peneumoniae* strain	2-butanone [mg/L]	2,3-butanediol [g/L]	acetoin [g/L]	lactate [g/L]	OD600
**wildtype**	ND	20.8± 1.6	2.8± 0.4	3.8± 0.6	4.5± 0.6
**KODDH**	111.2 ± 12.1	18.4± 1.2	2.8± 0.4	2.8± 0.4	4.2± 0.2
**LBDDH**	172.3 ± 23.2	17.8± 1.6	3.8± 0.7	3.2± 0.6	4.4± 0.2
**SEDDH**	94.5 ± 13.3	19.5± 0.9	2.8± 0.4	2.5± 0.8	3.8± 0.3

ND: not detectable

### Improvement of 2-butanone production by deletion of *ldhA* gene of *Klebsiellia pneumoniae*

As illustrated in Table 3, lactate is one of the major byproducts for 2, 3-butanediol and 2-butanone production. Lactate is normally toxic for cellular metabolism. To evaluate the effect of lactate for 2-butanone production, the plasmid pHSG298-lbpdu containing diol dehydratase and its activator from *L*. *brevis* was introduced into *K*. *pneumoniae* LDH 526, a mutant strain of *K*. *pneumoniae* HR526 with the deletion of *ldhA* gene [15]. The production of 2-butanone by *K*. *pneumoniae* LDH526/pHSG-lbpdu was increased to 246 ± 15 mg/L, which is 40% higher than that by *K*. *pneumoniae* HR526/pHSG-lbpdu (Table 4). The production of 2, 3-butanediol was increased by 12% while the production lactate was reduced by 92% (Table 4). The results indicated that knockout of *ldhA* gene encoding lactated dehydrogenase was favorable for 2-butanone production.

**Table 4:** 2-Butanone production by *K*. *peneumoniae* HR526/pHSG-lbpdu and *K*. *peneumoniae* LDH 526/pHSG-lbpdu.

*K*. *peneumoniae* strain	2-butanone [mg/L]	2,3-butanediol [g/L]	acetoin [g/L]	lactate [g/L]	OD600
**HR526/pHSG-lbpdu**	172.2 ± 23.2	17.8± 1.6	3.8± 0.7	3.2± 0.6	4.4± 0.2
**LDH526/pHSG-lbpdu**	246.4 ± 15.1	19.9± 2.4	3.2± 0.5	0.3± 0.1	3.9± 0.2

### Effect of coenzyme B12 for 2-butanone production

**Fig 2:**
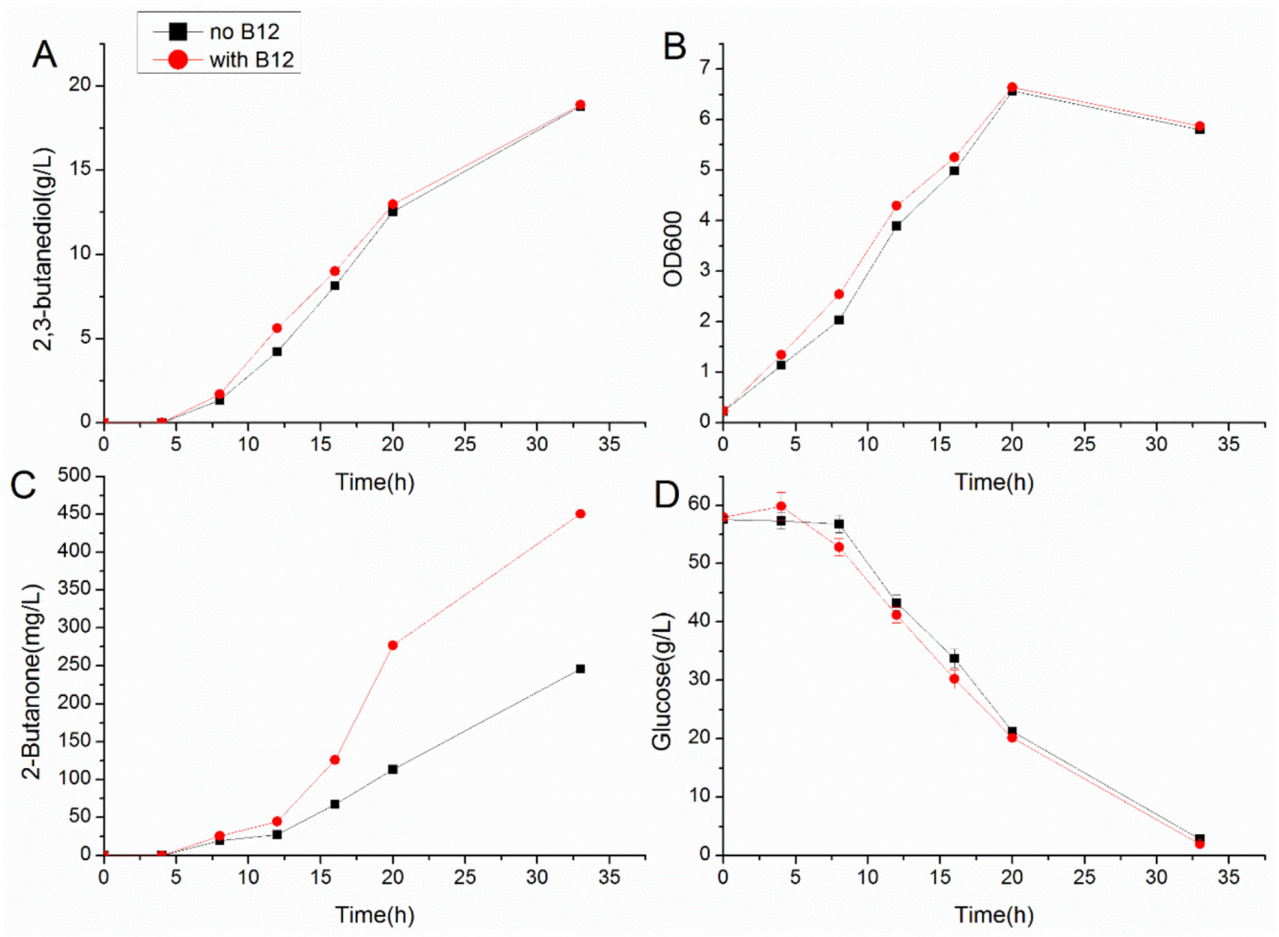
Effect of coenzyme B12 addition for 2-butanone production by *Klebsiella pneumoniae* LDH526/pHSG-lbpdu. (A) 2,3-butanediol production; (B) Cell growth; (C) 2-butanone synthesis; (D) glucose consumption. The cells were cultivated in 250 ml shake flask with 100 ml of fermentation medium with (1 μM) or without coenzyme B12 at 37°C, 250 rpm.

All of the glycerol dehydratases and diol dehydratases used in this study are coenzyme B12-dependent. Although *K*. *pneumonia* can natively produce coenzyme B12, the amount of coenzyme B12 under the culture condition may be limited. To test this hypothesis, 1 μM coenzyme B12 was added into the standard medium for the culture of *K*. *pneumoniae* LDH526/pHSG-lbpdu. As shown in Fig 2, with the addition of coenzyme B12, the production of 2-butanone increased to 450 mg/L, indicating that the additional supplement is beneficial for 2-butanone production under current condition. The addition of coenzyme B12 but did not change the production of other metabolites (Fig 2). It was reported that the synthesis of coenzyme B12 in *K*. *peneumoniae* was inhibited under aerobic condition [19]. With the further optimization of culture condition such as adjusting the stirring speed, we suppose that the addition of coenzyme B12 may be eliminated. This strategy has been previously used for 3-hydroxypropinate production in *K*. *peneumoniae* [20].

## Discussion

Production of bulk chemicals using renewable bioresources is an important goal of sustainable bioeconomy. Although large efforts have been made during the past decades, the production of non-natural metabolites by biological process are still highly challenged. One of the key obstacles is the lack of corresponding enzymes to catalyze the designed non-natural pathways. Screening of natural enzymes or protein engineering are the two major approaches to identify or design new catalytic activities [3–5]. 2, 3-Butanediol is an important product that can be efficiently generated during the fermentation of glucose by *K*. *pneumoniae*. It is also an important byproduct during the fermentation of glycerol by *K*. *pneumoniae*. However, the application of 2, 3-butanediol has been well explored. In this study, a non-natural metabolic pathway was built for 2-butanone production by the extension of the 2, 3-butanediol synthesis pathway. 2-Butanone is a very important bulk chemical with wide applications. Currently, the production of 2-butanone worldwide is exclusively by petrochemical route. There is no natural enzyme which can efficiently catalyze the dehydration of 2, 3-butanediol. Enzyme characterization showed that both glycerol dehydratases and diol dehydratases can catalyze the dehydration of meso-2, 3-butanediol although the catalytic efficiency is 2-order lower than the native reaction. Diol dehydratase generally showed better catalytic efficiency for meso-2, 3-butanediol dehydration than glycerol dehydratase. Enhancing the precursor supply by knocking out the *ldhA* gene and with the addition of coenzyme B12, *K*. *pneumoniae* can accumulate 2-butanone up to 450 mg/L. The production is still very low for industrial application. This is mainly due to the low activity of natural enzymes toward the dehydration of 2, 3-butanediol. Protein engineering to increase the enzyme activity and specificity is important for the further improvement of 2-butanone production. This part of work is ongoing. However, the results here already lays the basis for the development of *K*. *pneumoniae* for the biological production of 2-butanone. Moreover, this metabolic pathway can be further extended to 2-butanol, another important chemical that can be used as biofuel [21].

## Supporting Information

S1 Table Primers used for the pET28a-derived plasmids. (DOCX)

S2 Table Primers used for the construction of pHSG298-derived plasmids. (DOCX)

S3 Table The sequence accession numbers of glycerol dehydratases and diol dehydratases used in this study. (DOCX)
