# Supplementary material for: Metabolic Engineering of Klebsiella pneumoniae for the Production of 2-Butanone from Glucose
Source: PLoS One. 2015 Oct 14;10(10):e0140508. doi: 10.1371/journal.pone.0140508 (PMC4605612; doi:10.1371/journal.pone.0140508)
Supplement: S1 Table — (DOCX) [file pone.0140508.s001.docx]

**S1 Table. Primers used for the pET28a-derived plasmids.**

| Name | | Sequence | Purpose |
| --- | --- | --- | --- |
| kopdu-F | GCAAATGGGTCGCGGATCCGAATTCATGAGATCGAAAAGATTTGAAGCAC | | To amplify *pduCDE* of  *Klebsiella oxytoca* |
| kopdu-R | GGTGCTCGAGTGCGGCCGCAAGCTTTTAATCGTCGCCTTTGAGTTTTTTACG | |  |
| lbpdu-F | GCAAATGGGTCGCGGATCCGAATTCATGAAACGTCAAAAACGATTTGAAG | | To amplify *pduCDE* of  *Lactobacillus brevis* |
| lbbdu-R | GGTGCTCGAGTGCGGCCGCAAGCTTCTAGTTATCACCCTTCAGCTTCTTACG | |  |
| sepdu-F | GCAAATGGGTCGCGGATCCGAATTCATGAGATCGAAAAGATTTGAAGCAC | | To amplify *pduCDE* of  *Salmonella enterica* |
| sepdu-R | GGTGCTCGAGTGCGGCCGCAAGCTTTTAATCGTCGCCTTTGAGTTTTTTACG | |  |
| kpgld-F | GCAAATGGGTCGCGGATCCGAATTCATGAAAAGATCAAAACGATTTGCAGTAC | | To amplify *gldABC* of  *Klebsiella pneumoniae* |
| kpgld-R | GGTGCTCGAGTGCGGCCGCAAGCTTTTAGCTTCCTTTACGCAGCTTATG | |  |
| cfgld-F | GCAAATGGGTCGCGGATCCGAATTCATGAGAAGATCAAAACGATTCGAAG | | To amplify *gldABC* of *Citrobacter freundii* |
| cfgld-R | GGTGCTCGAGTGCGGCCGCAAGCTTTCACTGGCTGCCTTTACGCAG | |  |
| lrgld-F | GCAAATGGGTCGCGGATCCGAATTCATGAAACGTCAAAAACGATTTGAAG | | To amplify *gldABC* of  *Lactobacillus reuteri* |
| lrgld-R | GGTGCTCGAGTGCGGCCGCAAGCTTTTAGTTATCGCCCTTTAGCTTCT | |  |
|  |  | |  |
