# Supplementary material for: Metabolic Engineering of Klebsiella pneumoniae for the Production of 2-Butanone from Glucose
Source: PLoS One. 2015 Oct 14;10(10):e0140508. doi: 10.1371/journal.pone.0140508 (PMC4605612; doi:10.1371/journal.pone.0140508)
Supplement: S2 Table — (DOCX) [file pone.0140508.s002.docx]

**S2 Table. Primers used for the construction of *pHSG298-*derived plasmids.**

| Name | | Sequence | Purpose |
| --- | --- | --- | --- |
| Kopdu2-F | ctatgacatgattacgaattAAGGAGATATACCATGAGATCGAAAAGATTTGAAGCAC | | To amplify *pduCDEGH* of  *Klebsiella oxytoca* |
| Kopdu2-R | tgcctgcaggtcgactctagTTATTCATCCTGCTGTTCTCC | |  |
| Lbpdu2-F | ctatgacatgattacgaattAAGGAGATATACCATGAAACGTCAAAAACGATTTGAAG | | To amplify *pduCDEGH* of  *Lactobacillus brevis* |
| Lbbdu2-R | tgcctgcaggtcgactctagCTAATCTAATGTCTTAAAGGGCAC | |  |
| Sepdu2-F | ctatgacatgattacgaattAAGGAGATATACCATGAGATCGAAAAGATTTGAAGCAC | | To amplify *pduCDEGH* of  *Salmonella enterica* |
| Sepdu2-R | tgcctgcaggtcgactctagTTAAGCATGGAGATCCCGAA | |  |
