# Supplementary material for: Metabolic Engineering of Klebsiella pneumoniae for the Production of 2-Butanone from Glucose
Source: PLoS One. 2015 Oct 14;10(10):e0140508. doi: 10.1371/journal.pone.0140508 (PMC4605612; doi:10.1371/journal.pone.0140508)
Supplement: S3 Table — (DOCX) [file pone.0140508.s003.docx]

**S3 Table. The sequence accession numbers of glycerol dehydratases and diol dehydratases used in this study.**

| **Enzyme** | **Uniprot Acession No** |
| --- | --- |
| Diol dehydratase from *Klebsiella oxytoca* M5al | K6KSK5 |
| Diol dehydratase from *Lactobacillus brevis* ATCC 367 | Q03Q30 |
| Diol dehydratase from *Salmonella enterica* ATCC 700720 | P37450 |
| Glycerol dehydratase from *Klebsiella pneumoniae* MGH78578 | A6TEA0 |
| Glycerol dehydratase from *Citrobacter freundii* ATCC 29229 | D4BJJ6 |
| Glycerol dehydratase from *Lactobacillus reuteri* DSM20016 | A5VMB2 |
